# Consumption of antibacterial molecules in broiler production in Morocco

**DOI:** 10.1002/vms3.89

**Published:** 2018-01-08

**Authors:** Naoufal Rahmatallah, Hicham El Rhaffouli, Idriss Lahlou Amine, Yassine Sekhsokh, Ouafaa Fassi Fihri, Mohammed El Houadfi

**Affiliations:** ^1^ Avian Pathology Unit Department of Pathology and Veterinary Public Health Agronomy and Veterinary Institute Hassan II Rabat Morocco; ^2^ University Mohammed V Souissi Mohammed V Military Teaching Hospital Research and Biosafety Level 3 Laboratory Rabat Morocco

**Keywords:** Antibacterial consumption, antimicrobials, broilers, prescription

## Abstract

Monitoring the use of antibacterial agents in food‐producing animals is crucial in order to reduce antimicrobial resistance, selection and dissemination of resistant bacterial strains, and drug residues in the animal food products. The broiler production sector is considered a great consumer of antibacterials and incriminated in the rise of antimicrobial resistance level in zoonotic bacterial pathogens such as *Escherichia coli*,* Salmonella* and *Campylobacter*. Following recommendations from the OIE and WHO, a survey was conducted about the use and consumption of several antibacterial agents in Moroccan broiler flocks. More than 5 million broilers were randomly surveyed at the prescriber level, that is, via the veterinary clinics involved in their health management. The results showed that 93% of the flocks received at least one antibacterial treatment of minimum 3 days duration. Enrofloxacin, colistin and trimethoprim/sulphonamides were the most used antibacterials followed by oxytetracycline, florfenicol and amoxicillin. Oxytetracycline, enrofloxacin and colistin were overdosed in most of the administration, while amoxicillin and the combination of trimethoprim/sulphonamides were under‐dosed. The total amount of antibacterial consumed in the survey was 63.48 mg/kg and the Animal Level of Exposure to Antimicrobials (ALEA) was 94.45%. The reasons for this frequent use were related mainly to the poor quality of broiler production management. Chicks and animal feed provided to producers were of variable quality. Management of rearing stock density was often poor and biosecurity inadequate, and broilers were challenged by a high prevalence of infectious diseases.

## Introduction

The excessive use of antimicrobials in food‐producing animals rearing has raised serious concerns about the selection and transfer of multi‐resistant zoonotic foodborne pathogens of bacterial origin to human populations (Wegener 2003). In addition, the use of drugs without respect to withdrawal periods constitutes a threat to consumers with the persistence of residues in animal products (Salama *et al*. 2011; Darwish *et al*. 2013). Another concern is related to the contamination of water and environment with chemical residues. The persistence of antimicrobials in manure and the use of old litter in soil fertilization permits the leakage of drugs into wells and surface water (Phuong Hua *et al*. 2011; Meyer *et al*. 2013; Milic *et al*. 2013; Awad *et al*. 2014).

In order to limit these concerns, the World Health Organisation (WHO) in collaboration with the Food and Agriculture Organisation (FAO) and the Office International des Epizooties (OIE) recommend banning the use of antimicrobials as growth promoters and called for more controls for the therapeutic and prophylactic administration of antibacterial drugs to food animals (OIE, 2003; Collignon *et al*. 2009; Aidara‐Kane 2012; WHO, 2014).

In Morocco, the last 5 years have seen tremendous increase in poultry production. The total production of poultry meat jumped from 320 000 tons in 2006 to 610 000 tons in 2015 (Fisa, 2015). The broiler industry is the principal provider of animal protein making up 54% of total consumed meat. The broiler industry in Morocco has more than 7000 approved broiler units. The characteristics of broiler growing permit the production of pullets at the weight of 2.2 kg, at a mean of 42 rearing days and a conversion rate around 1.9 (Barkok 2007). However, the sector is poorly regulated due to the dominance of live market: 90% of broilers are sold live, with only 10% of broiler production processed in industrial slaughterhouses [Fédération Interprofessionnelle du Secteur Avicole (FISA) 2015]. The Moroccan authorities adopted a law (49.99) in 2006 following the worldwide crisis of avian influenza in order to regulate the poultry sector (Loi 49–99; 2002). Under the remit of the 49.99 law, all poultry producers are obliged to keep documents concerning the rearing, health status and treatments of their flocks (ONSSA, 2015).

Moroccan producers and veterinary practitioners involved in the broiler production are regular users of antimicrobials, and it is well known that the use of antimicrobials (either in therapeutic or preventative ways) has become a routine measure in order to ensure economic profitability for the broiler producers (Landers *et al*. 2012). In addition, antibacterial growth promoters (AGPs) are still permitted in Morocco and are used under veterinary prescription regularly by feed manufacturers. These AGP include arsenicals, antibiotics like virginiamycin, bacitracin, oxytetracycline, tylosin and coccidiostats. However, previous studies have shown treatment failures coupled with reports of increased antimicrobial resistance (Filali *et al*. 1988; Amara *et al*. 1995).

In addition, although Moroccan authorities have had a surveillance programme for some antimicrobials used in poultry, such as chloramphenicol and gentamicin since 2001 (ONSSA, 2015), they do not perform proficient monitoring for antimicrobial use and consumption in food animals compared to the DANMAP (2016) (Danish Programme for surveillance of antimicrobial consumption and resistance in bacteria from animals) and GERMAP (2016) (German programme for monitoring the consumption of antimicrobials and the extent of resistances against antimicrobials in human and veterinary medicine) programmes in Europe or the NAHMS (2016) (National Animal Health Monitoring System) in the United States. These programmes are responsible for the collection, analysis and publication of data on sales and consumption of antimicrobials in livestock and food animals [Chauvin *et al*. 2008; Merle *et al*. 2012; Food and Drug Administration (FDA) 2015a,b]. Furthermore, there were no reported studies concerning the use and consumption of antimicrobials in the broiler production in Morocco. Therefore, it is important to document the level of antimicrobial use in broilers given the fact that it is the major source of meat and that poultry sector is considered as the major destination of commercialized antimicrobials.

The purpose of this study is to report the quantification and patterns of use of antimicrobials in the Moroccan broiler sector based on the results of a survey conducted at the prescribers' level, that is, the private veterinarians involved in the health management of broiler production.

## Materials and methods

### Survey design and sampling

In Morocco, there are approximately 35 major veterinary clinics involved in the health management of poultry units. Among these, 20 clinics have more than 80% of the activity for the health management of poultry. The remaining 15 clinics only count poultry activity at the level of 35–60% (Bennani 2016). Broiler producers are obliged to have management contracts with the veterinary clinics involved in poultry disease activity according to the 49.99 Moroccan law (Loi 49–99; 2002) (ONSSA, 2015). We selected 20 veterinary clinics in the main geographical areas of poultry production and randomly chose records for at least 250 000 broilers raised from different flocks to investigate antimicrobial use. Randomization was done by assigning numbers to broiler flocks and selecting the first ascending list of random numbers in Excel spreadsheet. Twenty veterinary clinics were contacted, 12 responded and 8 were not willing to participate. From the 12 responding clinics, two sets of data were discarded because one included turkey production and the other only provided data about two flocks. The final data included 280 broiler units ranging from 5000 to 105 700 birds per flock.

In order to assess the sampling power of our study, we took into account 80% desired power, 1.96 for statistical significance and 5% error margin for sample size determination. The statistical calculation provided a sample size requirement of 245 flocks. In the survey, we collected data from 280 flocks which exceed sample size requirements.

### Data collection

The survey included data of over 250 000 broilers produced during a period of 2 years. The veterinarians were asked to give information concerning the prescription of antimicrobial treatments for the treatment of flocks; question about AGPs were not included in the survey. We sent the veterinary clinics a questionnaire in which they were asked to report all treatment records of the different flocks reared during the study period. The questionnaire also included indications for treatments, information about the flocks and other data such as susceptibility tests as they are not usually reported in the producers' treatment records. The data about the broiler flocks included the following: total number of birds in the flock, season of rearing, age at treatment and reasons for treatments. The information about the prescription and delivery of antibacterials concerned the indication for treatments, presence of bacteriological analysis (isolation of pathogens and antimicrobial susceptibility tests), prescribed antimicrobial substances, concentration of active substances, posology, delivered amount of antibacterials and follow‐up after the treatment. The following indications for treatments were included in the questionnaire: prevention against omphalitis, prevention against chronic respiratory diseases (CRD) and treatments for omphalitis, enteritis, colibacillosis, coccidiosis, feet problems or arthritis.

### Data analysis

The collected data were entered in an Excel spreadsheet (Microsoft Corporation, Redmond, Washington, USA) for further statistical analysis that include descriptive statistics [mean, standard deviation (SD), maximum and minimum (max, min) observations] for flocks and treatments, and ANOVA analysis. The volumes of antimicrobials administered were converted to mg of active substance per kg live weight. Calculations for the frequency of use as well as the consumed amount of the different active substances, the differences between seasons, the age of administration for the broiler flocks and the differences in the distribution of treatment causes were performed.

In order to assess the correctness of prescription, the following weight indicators were chosen: the defined daily dose (DDD) that is defined as the nationally determined average maintenance dose per day and per kg chicken of a specific drug (Chauvin *et al*. 2001; Jensen *et al*. 2004). For poultry, the DDD was estimated based on the dosages mentioned in the Moroccan Dictionary of Veterinary Drugs (2015) (http://www.dmv-maroc.com/) and on the drug's instruction leaflet. Live weights of chicken were taken from weight standards table of Ross breeds. The used daily dose (UDD) describes the amount of active substance actually administered to the animals in mg/kg (Grave *et al*. 2004). The UDD was calculated by dividing the amount of antimicrobial compound administered (mg) by the number of broilers time the average weight at treatment to define a standard treated bird (Timmerman *et al*. 2006). The UDD/DDD ratios were calculated as a way to assess the correctness of dosage. Ratios between 0.8 and 1.2 were considered as correct dosing. Values <0.8 and >1.2 were considered to be under‐dose and overdose, respectively (Timmerman *et al*. 2006). The consumption of antimicrobials was calculated by dividing the total amount of used antibacterial in mg by the average weight at slaughter for all the surveyed flocks. The Animal Level of Exposure to Antibacterials (ALEA; which is an exposure data used in France since 1999) was calculated by dividing the treated live weight by the total live weight of surveyed flocks (Anses report on sales survey, 2015).

## Results

The survey included 280 flocks containing 5 658 600 broilers chicks reared between January 2014 and December 2015. The mean size of the flocks was 25 720 with minimum size of 5000 and the maximum of 105 700 birds per flock.

### Antimicrobial treatments

A total of 484 antimicrobial treatments (for entire flocks) were recorded; 222 were administered during the first week of age, 14 in the second week, 64 in the third week and 92 treatments for both weeks 4 and 5.

The majority of flocks (92.5%) received at least one treatment; 31.67% were treated twice, 16.7% received three treatments and 5% were given four antimicrobial treatments. Only 21 flocks totalizing 314 000 broilers (7.5%) did not receive any antimicrobial treatments.

According to these data, the number of antimicrobial treatments ranged from 0 to 5 with each flock of broiler receiving a median of 1.73 (±SD 1.01) antimicrobial treatments during its production life. The ALEA was 94.45%.

Concerning susceptibility testing, 32% of antimicrobial treatments were given on the basis of bacteriological investigation and antimicrobial susceptibility tests, whereas 68% were given empirically. The susceptibility tests were essentially done on *Escherichia coli* through agar diffusion methods using antibacterial discs according to EUCAST guidelines (EUCAST, 2016). The reported reasons for treatments are shown in Table 1.

**Table 1:** Distribution of antimicrobial use related to reasons for treatment at different ages

Reasons for treatments	Number of treatments	%
Prophylaxis at 1 week*	206	42.56
Prophylaxis at 2 weeks†	4	0.83
Prophylaxis at 3 weeks†	15	3.10
Colisepticaemia	123	25.41
Non‐specified enteritis	82	16.94
CRD	77	15.91
Omphalitis	15	3.10
Unspecified	9	1.86

*Prophylaxis at first week means using antimicrobials to prevent early chick mortality. †Prophylaxis at 2 and 3 weeks means using antimicrobials to prevent chronic respiratory diseases.

The reasons for antibacterial treatments were for both preventative and therapeutic purposes. Prevention against omphalitis and early chick mortality was the reason for 42.56% of treatments. Prevention against respiratory diseases [especially chronic respiratory disease (CRD) associated with mycoplasma] accounted for 3.93%; 3.10% of treatments administered in week 3 and 0.83% at week 2. Concerning therapeutic treatments, colisepticaemia was considered as the first reason for treatment by 25.41%. Treatments against enteric disorders were the second most common reason (16.94%) for therapeutic treatments. CRD was the third most common indication at 15.91%.

It is worth noting that omphalitis was treated in 3.14% of cases despite the prior use of preventative treatments. Also, it was noted that some veterinarians reported more than one reason for antimicrobial treatment such as enteric disorders plus respiratory diseases and prevention against chick mortality plus omphalitis.

**Figure 1:**
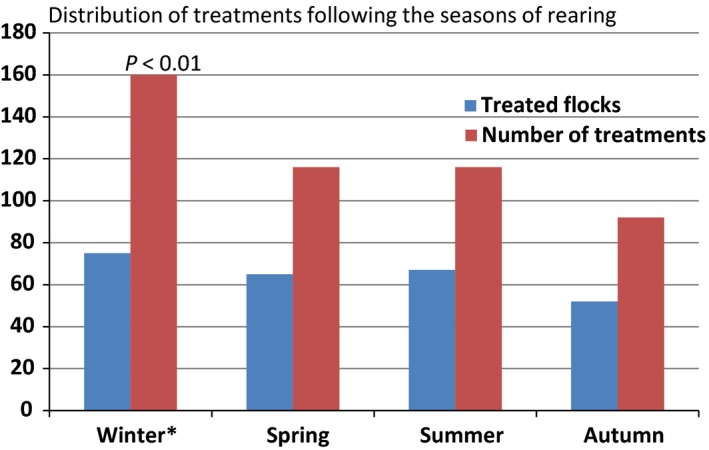
Distribution of antimicrobial uses per flock during different seasons.

Concerning the effect of the seasons on the distribution of treatments, there were significant differences by ANOVA in the seasonal distribution of antimicrobial treatments, noting that the majority of broiler flocks were raised in houses with no climate control systems. Winter was the season when broiler flocks received most treatments; 143 treatments for 44 surveyed flocks with a significant statistical difference (*P* < 0.01) compared to other seasons (Fig. 1). In summer, there were 133 treatments for 73 surveyed flocks, but in autumn and spring, the recorded treatments were 99 and 113, respectively, for 60 and 64 reared flocks and no statistical differences were noted.

### Consumption of antimicrobial components

The survey revealed that enrofloxacin was the most commonly used antimicrobial with 108.6 kg of active substance for treating 2 904 800 broilers. Colistin was second with 1 164 060 millions of IU of colistin sulphate used equivalent to 56.78 kg of active molecule distributed either by administration in drinking water for 2 305 000 birds or intramuscular injection for 1 100 000 broilers.

Table 2 summarizes the different antimicrobials used as found in the survey, as well as their final amounts in g per active substance and the total number of birds treated.

**Table 2:** Use of the active antibacterials identified in the survey

Antibacterial	Number of treatments^a^	Consumed amount in kg of active substance	Number of birds treated^a^	Consumption (mg/kg)
Enrofloxacin	117	108.6	2 904 800	18.69
Colistin	139	56.78	3 405 000	8.40
Amoxicillin	31	66.74	765 300	43.60
Sulphonamides	85	64.38	1 739 200	18.51
Trimethoprim	85	13.463	1 739 200	3.87
Florfenicol	46	110.45	1 034 000	53.41
Oxytetracycline	49	263.19	1 440 000	127.27
Lincomycin	13	2.38	191 000	0.83
Spectinomycin	13	4.77	191 000	12.48
Doxycycline	6	20.50	137 000	53.66
Tylosin	4	2.55	61 000	9.31
Erythromycin	2	0.60	53 200	4.92
Fosfomycin	2	3.00	50 000	28.20
Neomycin	6	0.33	116 000	3.25
Total		717.72	5 658 600^a^	63.48^a^

a The number of treatments and the number of treated birds are higher than the total number of surveyed broilers because most flocks received more than one antibiotic treatment.

The UDD and DDD were distributed differently according to the antibacterial used and the age of treatment. The distribution ranged between 4.25 for oxytetracycline and 0.68 for sulfadiazine–trimethoprim (Table 3).

**Table 3:** UDD/DDD ratios for the main antibacterials in the survey

Antibacterial	Number of treated broilers	Mean day at treatment (no.)	Total live weight (kg) at the treatment day	DDD (g)	UDD (g)	UDD/DDD ratio
Enrofloxacin	2 208 340	Day 2 (67) + Day 25 (16)	758 690	7586.9	16 270	2.14
Combination of enrofloxacin + colistin	739 000	Day 1 (2) + Day 24 (32)	763 698	7636.98	30 280	3.96
				65 335 MIU	252 800 MIU	3.87
Colistin (oral)	1 538 000	Day 2 (42) + Day 17 (52)	676 780	50 758 MIU	128 000 MIU	2.52
Colistin (injection)	1 160 000	Day 26	1 485 120	74 256 MIU	86 218 MIU	1.16
Florfenicol	1 034 000	Day 1 (2) + Day 23 (45)	1 113 034.5	28 030.94	27 100	0.97
Amoxicillin	585 400	Day 27	817 580	15 629.2	11 380	0.73
Sulfadimethoxine + TMP^a^	1 365 000	Day 3 (63) + Day 23 (7)	326 995	79 445.28	67 353.75	0.85
Sulfadiazine + TMP^a^	314 300	Day 4 (9) + Day 25 (5)	258 318	77 495.4	53 000	0.68
Oxytetracycline	1 075 000	Day 24	1 307 600	13 076	55 541.67	4.25

a TMP, trimethoprim.

The total amount of antimicrobial drugs per kg of produced broiler meat in this survey was 717.72 kg of all active components for 11 317 200 kg equivalent of broiler at the age of slaughter, which was equivalent to 63.48 mg of active antimicrobial component per kg of produced broiler meat.

## Discussion

The methodology of the survey took its basis from the 2012 Terrestrial Animal Health Code issued by the OIE (2014). This manual describes the nature of data recovery from records concerning the total number of treated animals, their estimated live weight, the total amount of active ingredients of the used antimicrobials and other information such as the age of treatment, the duration and the season where the antimicrobial treatment occurred.

The survey focused on data related to antimicrobial prescriptions and delivery from private veterinary practices, that is, the prescribers, involved in the health care of broilers in Morocco. Indeed, veterinarians are considered to be the most reliable way to obtain precise information on the nature of prescriptions, dosage, number of treatment days, age of birds at the time of treatment and the amount of antimicrobials delivered [Chauvin *et al*. 2001; World Organisation For Animal Health (OIE) 2014]. According to Moroccan law on poultry production, veterinary practitioners supervising the broiler operations must keep records at the farm (ONSSA, 2015) the prescriptions, the dosage and the delivery of antibacterials. These data are difficult to obtain from either broiler producers or the veterinary pharmaceutical industry.

The results of this survey showed that the antimicrobials are commonly administrated to broilers. Indeed, 93% of the investigated flocks received treatments lasting at least 3 days during the period of rearing. The reasons for this regular consumption are multiple: poor rearing practices, low‐quality inputs especially day old chicks and feed, veterinary' practices and lack of efficient control by authorities responsible for the safety of food animal production.

First of all, the structure of the poultry industry makes it difficult for broiler producers to install environmentally controlled plants (Barkok 2007). The costs of these buildings is still high in Morocco and do not allow a rapid return on investments for broiler producers. The shrinking profit margins registered during the past years could be an important factor. In addition, there are no subsidies or financial support provided for producers investing in controlled houses. Therefore, the broiler flocks are continually under the pressure of seasonal factors of cold and heat coupled with poor biosecurity measures. This situation frequently drives the use of antibacterial agents to treat flocks. In fact, the use of traditional housing systems may explain the observed statistical difference in the density of treatments between seasons. The increase in treatments during winter may be because of low temperatures and stocking density stress in the houses resulting in poor ventilation.

Poor ventilation and litter quality are major sources of distress to broilers. When they are associated with high density (that the majority of producers use to elevate the temperature in winter in the absence of environmentally controlled houses), they could predispose to respiratory and digestive problems that the producers then try to solve with antimicrobial treatments (Manning *et al*. 2007).

Another important factor in Morocco is the spread and the high pressure of some viral pathogens such as infectious bronchitis virus (Fellahi *et al*. 2015) and the contamination of parental flocks with *Mycoplasma synoviae* (Nassik *et al*. 2014). The viral pathogens lead to more secondary *E. coli* infections which further increase the use of antimicrobial treatments (Barnes *et al*. 2003).

The poor quality of day‐old chicks can be considered as the major factor for the use of antimicrobials in broilers. The high prevalence of omphalitis and yolk infections leads to a systematic approach of using antimicrobials in chicks during the first week of life. This might explain why 41% of flocks received prophylactic treatments in the first week of age. These findings are different from the Chinese study of Krishnasamy *et al*. (2015) which found that 75% of antimicrobial treatments in broilers are administered during the grower stage. However, similar results to ours are reported in the Vietnamese study of Carrique‐Mas *et al*. (2015) with 84% of antimicrobials use in meat‐type chicken for prophylactic purposes.

Poultry feed in Morocco is of variable quality and is believed to be a major cause of enteric disorders, thereby increasing the use and consumption of antimicrobials. Indeed, a study about antimicrobial resistance reported an increase in amoxicillin level of resistance correlated with enteric disorders (Rahmatallah *et al*. 2017). Non‐specific enteritis and necrotic enteritis are treated in the first place by amoxicillin followed by oxytetracycline. Coccidiosis is treated by sulphonamides.

The veterinary practitioners responsible for broiler flocks' health management can also be considered as actors in this consumption of antimicrobials. The findings of overdosing with antibacterials such as oxytetracycline (UDD/DDD ratio of 4.25) and enrofloxacin (ratio of 2.14) question the prescription practices of the veterinarians. This might suggest that the prescribers are contributing to the over‐usage of antimicrobials. Veterinarians should apply good prescription practices for antimicrobials by stressing the need for an accurate diagnosis, the appropriate choice of antimicrobials, the best dose prescription and the use of alternative such as probiotics, organic acids or phytotherapy (Passantino 2007; Hume 2011). They have to educate broiler producers that antimicrobials are only used in case of extreme necessity and have to restrain themselves from over‐using prophylactic treatments (Aidara‐Kane 2012). They should also put more emphasis on the application of biosecurity measures and good practice in hygiene and rearing management (Clark *et al*. 2012).

The survey revealed that the prescription of treatments was for both prophylactic and therapeutic purposes in equal measure. This indicates that rearing broilers in Morocco is subject to many disease challenges. Veterinarians explained the use of prophylactic treatments by early chick mortality caused by egg yolk infection and high prevalence of respiratory diseases at the beginning of the third week of age. For therapeutic administrations, the main reasons were for controlling CRD (77 treatments) often complicated with colibacillosis (123 cases), enteritis (88 treatments) and other unspecified diseases such as arthritis (7 cases).

Concerning the frequencies of antimicrobial use in this survey, the results show that the most commonly used antimicrobials were colistin (27.85% of treatments), enrofloxacin (23.44%), the association of trimethoprim–sulphonamide compounds (17.03%) and oxytetracycline (9.81%).

These results differ from those found by the ANSES (France) and NAHMS (USA) reports where oxytetracycline was the most used antimicrobial with 39.34% and 43% of total antimicrobial sales in France and in the United States, respectively (Bondt *et al*. 2013; Anses, 2015; FDA, 2015a,b). Colistin was used at the rate of 6.58% in France and 2% (among other antibacterials) in the United States. Enrofloxacin was used in 0.63% of treatments in France and <1% in the United States (Anses, 2015; FDA, 2015a,b).

However, the French and American data concerned all animal species and were based on pharmaceutical sales. Also, AGPs are still used in the United States, although prohibited in France. In addition, enrofloxacin use has been banned for poultry in the United States since 2005 (Nelson *et al*. 2007; FDA, 2015b, final decision of the commissioner). One reason for these differences relates to the fact that 45% of antimicrobials used in food‐producing animals were distributed in the feed as the route of administration (FDA, 2015a,b), with tetracycline as the major compound. Also, 50% of tested *E. coli* in broilers in France were susceptible to tetracycline treatments (Anses Resapath, 2015), while more than 60% of *E. coli* tested in Morocco were resistant (Amara *et al*. 1995). Furthermore, recent reports show resistance in more than 90% of tested strains. Reasons are related to the excessive use of tetracycline as an AGP and frequently sold over the counter which has probably permitted the selection of resistant Gram‐negative organisms, especially *E. coli* (Rahmatallah *et al*. 2013, 2017).

The UDD/DDD ratio is a method for assessing the correctness of antimicrobial dosing (Persoons *et al*. 2012). In our survey, oxytetracycline was the most markedly overdosed drug with a UDD/DDD ratio of 4.25. Amoxicillin and trimethoprim–sulfadiazine were under‐dosed with ratios of 0.73 and 0.68, respectively. These results differ from the Belgian study of Persoons *et al*. (2012) who reported slight overdosing for amoxicillin and trimethoprim–sulphonamides with UDD/DDD ratio of 1.3 for both antimicrobials.

For oxytetracycline, the reasons of this overdosage seemed to be related to the prescribers' emphasis on its usage based on mg per litre of drinking water rather than a posology based on animal body weight. Regarding the excess of water consumption in birds especially in case of enteric disorders or heat stress (Morocco is a country with a hot climate), the dosing of the drug could therefore be overestimated. Furthermore, the delivery of antimicrobials is affected by the physicochemical properties of the water supply, especially for powder formulations. Several studies have demonstrated the ability of tetracycline to bind strongly to divalent metals, especially calcium and magnesium (Werner *et al*. 2006; Wammer *et al*. 2011). The well waters in Morocco are generally basic and contain significant traces of minerals (Belghiti *et al*. 2014) that impede the solubility of certain antibacterials, especially oxytetracycline which may mitigate the overdosing.

Of concern are the differences noted in UDD/DDD ratios related to the age at treatment. For example, colistin and enrofloxacin ratios ranged between 2.1 at the grower stage (17–28 days of age) to 12 at the starter age (generally the first week). The reasons for this difference were also related to the prescription dosage in mg per litre of drinking water which gave overdosage in comparison to prescription based on live body weight when broilers were small.

The amount of consumed antimicrobials in this survey was 63.48 mg per kg of produced broiler meat which might be considered a significant amount. Yet in France, 78.06 mg of antimicrobials per kg of poultry products was used (Anses, 2015), and in the United States usage of antimicrobials was estimated at 145 mg/kg (van Boeckel *et al*. 2015). A study of Chinese use of antimicrobials in food‐producing animals ranged the consumption between 301 and 796 mg/kg (Krishnasamy *et al*. 2015). However, comparison with these results is limited due to differences in data collection and methodology. In this study, data were obtained from prescribers and concerned only antimicrobials used in therapy or prophylactic purposes for broilers and not AGPs. In France, the consumption does not include AGP which are banned, but was assessed for all poultry products including several avian and anatidae species (broilers, layers, turkeys, quails, ducks, pigeons and goose), and the ratio was calculated based on the sales records of pharmaceutical companies (Anses, 2015). In the United States, both the consumption of AGPs (including coccidiostats and arsenicals) and therapeutic antimicrobials was based on sales records in order to give a total estimate of antimicrobial consumption (van Boeckel *et al*. 2015). The Chinese study was based on assumptions and mathematical modelling using data on the total amount of antimicrobials (including AGP and coccidiostats) sold for food animals and an estimation on poultry production at the time of the study (Krishnasamy *et al*. 2015).

## Conclusion

This study is the first attempt to monitor antimicrobial use and consumption in the Moroccan broiler production. It shows that antimicrobials are used frequently, and the amounts tend to approach those of developed countries. Many intervening players including producers, private veterinarians and sanitary authorities are identified in this survey as potential causes for this regular use. These stakeholders need to achieve a common sense approach to the importance of enhancing the quality of broiler production versus controlling measures for antimicrobial use and consumption. The national authority responsible for animal production and food animal products control should set‐up surveillance and monitoring systems for antimicrobial use and antibacterial resistance levels in food‐producing animals.

## Source of Funding

None.

## Conflicts of Interest

None declared.

## Contributions

N. Rahmatallah and H. El Rhaffouli conceived of the presented idea. N. Rahmatallah developed the theory and performed the computations. H. El Rhaffouli, I. Lahlou Amine, Y. Sekhsokhe and M. El Houadfi verified the analytical methods and supervised the findings of this work. All authors discussed the results and contributed to the final manuscript.

## Ethics in animal experimentation

No experimentations were undertaken using animals.

## References

[vms389-bib-0001] Loi n 49‐99 relative à la protection sanitaire des élevages avicoles, au contrôle de la production et la commercialisation des produits avicoles, promulguée par le dahir n 1‐02‐ 119 du 1 rabii Il 1423 (13 juin 2002) (BO n°5036 du 5 Septembre 2002, page : 901)

[vms389-bib-0002] Aidara‐Kane A. (2012) Containment of antimicrobial resistance due to use of antimicrobial agents in animals intended for food: WHO perspective. Revue scientifique et technique (International Office of Epizootics) 31, 277–287. 22849282 10.20506/rst.31.1.2115

[vms389-bib-0003] Amara A. , Ziani Z. & Bouzoubaa K. (1995) Antibiotic resistance of *Escherichia coli* strains isolated in Morocco from chickens with colibacillosis. Veterinary Microbiology 43, 325–30. 7785191 10.1016/0378-1135(94)00101-2

[vms389-bib-0004] Anses (2015). Sales survey of veterinary medicinal products containing antimicrobials in France 2015. https://www.anses.fr/fr/system/files/ANMV-Ra-Antibiotiques2015.pdf. (Accessed 17 September 2015)

[vms389-bib-0005] Anses, Resapath (2015). Rapport annuel du réseau d'épidémiosurveillance de l'antibiorésistance des bactéries pathogènes animales 2014. Available at: https://www.anses.fr/fr/system/files/ANMV-Ra-Antibiotiques2014.pdf. (Accessed 2 January 2016)

[vms389-bib-0006] Awad Y.M. , Kim S.C. , El‐Azeem S.A. , Kim K.H. , Kim K.R. , Kim K. *et al* (2014) Veterinary antibiotics contamination in water, sediment, and soil near a swine manure composting facility. Environmental Earth Sciences 71, 1433–40.

[vms389-bib-0007] Barkok A. . (2007) Revue du secteur avicole. Production system characteristics in FAO publications. Available at: http://ftp://ftp.fao.org/docrep/fao/011/ai377f/ai377f00.pdf. (Accessed 13 September 2014)

[vms389-bib-0008] Barnes H.J. , Vaillancourt J.P. & Gross W.B. (2003) Colibaccilosis In: Diseases of poultry. 11th edn, 631–656. (eds Saif Y.M., Barnes H.J., Glisson J.R., Fadly A.M., McDougald L.R. & Swayne D.E.), Iowa State Press Ames.

[vms389-bib-0009] Belghiti L. , Chahlaoui A. , Bengoumi D. & el Moustaine R. (2014) Effect of anthropic activities on the quality of subsoil waters in rural medium in the area of Meknes (Morocco). Larhyss Journal 17, 77–89.ISSN 1112‐3680

[vms389-bib-0010] Bennani H. . (2016) The actual situation of antibiotic use in poultry, Morocco. Veterinary thesis. Hassan II Agronomic and veterinary institute, Rabat.

[vms389-bib-0011] van Boeckel T.P. , Brower C. , Gilbert M. , Grenfell B.T. , Levin S.A. , Robinson T.P. *et al* (2015) Global trends in antimicrobial use in food animals. Proceedings of the National Academy of Sciences of the United States of America 112, 5649–54. 25792457 10.1073/pnas.1503141112 PMC4426470

[vms389-bib-0012] Bondt N. , Jensen V.F. , Puister‐Jansen L.F. & van Geijlswijk I.M. (2013) Comparing antimicrobial exposure based on sales data. Preventive Veterinary Medicine 108, 10–20. 22897857 10.1016/j.prevetmed.2012.07.009

[vms389-bib-0013] Carrique‐Mas J.J. , Trung N.V. , Hoa N.T. , Mai H.H. , Thanh T.H. , Campbell J.I. *et al* (2015) Antimicrobial usage in chicken production in the Mekong delta of Vietnam. Zoonoses and Public Health 62(suppl. 1), 70–78. 25430661 10.1111/zph.12165

[vms389-bib-0014] Chauvin C. , Madec F. , Guillemot D. & Sanders P. (2001) The crucial question of standardisation when measuring drug consumption. Veterinary Research 32, 533–543. 11777005 10.1051/vetres:2001145

[vms389-bib-0015] Chauvin C. , Querrec M. , Perot A. , Guillemot D. & Sanders P. (2008) Impact of antimicrobial drug usage measures on the identification of heavy users, patterns of usage of the different antimicrobial classes and time‐trends evolution. Journal of Veterinary Pharmacology and Therapeutics 31, 301–311. 18638290 10.1111/j.1365-2885.2008.00960.x

[vms389-bib-0016] Clark S. , Daly R. , Jordan E. , Lee J. , Mathew A. & Ebner P. (2012) The future of biosecurity and antimicrobial use in livestock production in the United States and the role of extension. Journal of Animal Science 90, 2861–2872. 22896737 10.2527/jas.2011-4739

[vms389-bib-0017] Collignon P. , Powers J.H. , Chiller T.M. , Aidara‐Kane A. & Aarestrup F.M. (2009) World Health Organization ranking of antimicrobials according to their importance in human medicine: a critical step for developing risk management strategies for the use of antimicrobials in food production animals. Clinical Infectious Diseases 49, 132–141. 19489713 10.1086/599374

[vms389-bib-0018] DANMAP (2016) Danish Programme for surveillance of antimicrobial consumption and resistance in bacteria from animals. Available from: https://www.danmap.org/(Accessed 2 January 2016)

[vms389-bib-0019] Darwish W.S. , Eldaly E.A. , El‐Abbasy M.T. , Ikenaka Y. , Nakayama S. & Ishizuka M. (2013) Antibiotic residues in food: the African scenario. Japanese Journal of Veterinary Research 61, S13–S22. 23631148

[vms389-bib-0020] EUCAST , (2016). EUCAST Disk Diffusion Method for Antimicrobial Susceptibility Testing. Available from: http://www.eucast.org/ast_of_bacteria/disk_diffusion_methodology/(Accessed 2 January 2016)

[vms389-bib-0021] Fédération Interprofessionnelle du Secteur Avicole (FISA) (2015). Production reports. Available from:http://www.fisamaroc.org.ma/index.php?option=com_content&view=article&id=65&Itemid=49. (Accessed on 13 September 2015)

[vms389-bib-0022] Fellahi S. , Ducatez M. , el Harrak M. , Guérin J.L. , Touil N. , Sebbar G. *et al* (2015) Prevalence and molecular characterization of avian infectious bronchitis virus in poultry flocks in Morocco from 2010 to 2014 and first detection of Italy 02 in Africa. Avian Pathology 44, 287–95. 25925561 10.1080/03079457.2015.1044422

[vms389-bib-0023] Filali E. , Bell J.G. , el Houadfi M. , Huggins M.B. & Cook J.K.A. (1988) Antibiotic resistance of *Escherichia coli* strains isolated from chickens with colisepticaemia in Morocco. Comparative Immunology, Microbiology and Infectious Diseases ll, 121–124. 10.1016/0147-9571(88)90027-6 3053022

[vms389-bib-0024] Food And Drug Administration (FDA) (2015a). FDA Annual summary report on antimicrobials sold or distributed in 2012 for use in food‐producing animals. http://www.fda.gov/AnimalVeterinary/NewsEvents/CVMUpdates/ucm416974.htm. (Accessed 2 January 2016)

[vms389-bib-0025] Food and Drug Administration (FDA) . (2015b). Final decision of the commissioner: withdrawal of approval of the new animal drug application for enrofloxacin in poultry. Rockville, MD: US FDA, 2005. Available at: http://www.fda.gov/animalveterinary/safetyhealth/recallswithdrawals/ucm042004.html. (Accessed 2 January 2016)

[vms389-bib-0026] GERMAP (2016). German programme for monitoring the consumption of antimicrobials and the extent of resistances against antimicrobials in human and veterinary medicine. Available from: https://www.bvl.bund.de/DE/Home/homepage_node.html (Accessed 2 January 2016).

[vms389-bib-0027] Grave K. , Kaldhusdal M.C. , Kruse H. , Harr L.M. & Flatlandsmo K. (2004) What has happened in Norway after the ban of avoparcin? Consumption of antimicrobials by poultry. Preventive Veterinary Medicine 62, 59–72. 15154685 10.1016/j.prevetmed.2003.08.009

[vms389-bib-0028] Hume M.E. (2011) Historic perspective: prebiotics, probiotics, and other alternatives to antibiotics. food safety symposium: potential impact of reduced antibiotic use and the roles of prebiotics, probiotics, and other alternatives in antibiotic‐free broiler production. Poultry Science 90, 2663–2669. 10.3382/ps.2010-01030 22010256

[vms389-bib-0029] Jensen V.F. , Jacobsen E. & Bager F. (2004) Veterinary antimicrobial‐usage statistics based on standardized measures of dosage. Preventive Veterinary Medicine 64, 201–215. 15325773 10.1016/j.prevetmed.2004.04.001

[vms389-bib-0030] Krishnasamy V. , Otte J. & Silbergeld E. (2015) Antimicrobial use in Chinese swine and broiler poultry production. Antimicrobial Resistance and Infection Control 28, 4–17. 10.1186/s13756-015-0050-y PMC4412119 25922664

[vms389-bib-0031] Landers T.F. , Cohen B. , Wittum T.E. & Larson E.L. (2012) A review of antibiotic use in food animals: perspective, policy, and potential. Public Health Reports 127, 4–22. 22298919 10.1177/003335491212700103 PMC3234384

[vms389-bib-0032] Manning L. , Chadd S.A. & Baines R.N. (2007) Key health and welfare indicators for broiler production. World's Poultry Science Journal 63, 46–62.

[vms389-bib-0033] Merle R. , Hajek P. , Käsbohrer A. , Hegger‐Gravenhorst C. , Mollenhauer Y. , Robanus M. *et al* (2012) Monitoring of antibiotic consumption in livestock: a German feasibility study. Preventive Veterinary Medicine 104, 34–43. 22115924 10.1016/j.prevetmed.2011.10.013

[vms389-bib-0034] Meyer E. , Gastmeier P. , Dejab M. & Schwab F. (2013) Antibiotic consumption and resistance: data from Europe and Germany. International Journal of Medicine Microbiology 303, 388–395. 10.1016/j.ijmm.2013.04.004 23727396

[vms389-bib-0035] Milic N. , Milanovic M. , Letic N.G. , Sekulic M.T. , Radonic J. , Mihajlovic I. & Miloradov M.V. (2013) Occurrence of antibiotics as emerging contaminant substances in aquatic environment. International Journal of Environmental Health Research 23, 296–310. 23067248 10.1080/09603123.2012.733934

[vms389-bib-0036] Moroccan Dictionary of Veterinary Drugs (2015). Available from: http://www.dmv-maroc.com/index.php? (Accessed on 15 September 2015)

[vms389-bib-0037] NAHMS the National Animal Health Monitoring System in the USA (2016). Available from: https://www.aphis.usda.gov/aphis/ourfocus/animalhealth/monitoring-and-surveillance/nahms/ (Accessed 2 January 2016)

[vms389-bib-0038] Nassik S. , Aboukhalid R. , Azzam F. , Rahmatallah N. , Lahlou‐Amine I. , Fassi‐Fihri O. & el Houadfi M. (2014) Detection of mycoplasma synoviae infection in broiler breeder farms of morocco using serological assays and real time PCR. Life Science Journal 8, 815–821.

[vms389-bib-0039] Nelson J.M. , Chiller T.M. , Powers J.H. & Angulo F.J. (2007) Fluoroquinolone‐resistant campylobacter species and the withdrawal of fluoroquinolones from use in poultry: a public health success story. Clinical Infectious Diseases 44, 977–980. 17342653 10.1086/512369

[vms389-bib-0040] ONSSA (2015). Office national de sécurité sanitaire des aliments. Requirements for poultry production. Available from http://www.onssa.gov.ma/fr/index.php?option=com_content&view=article&id=187&Itemid=126 (Accessed 10 March 2015).

[vms389-bib-0041] Passantino A. (2007) Ethical aspects for veterinarians regarding antimicrobial drug use in Italy. International Journal of Antimicrobial Agents 29, 240–244. 17204404 10.1016/j.ijantimicag.2006.09.023

[vms389-bib-0042] Persoons D. , Dewulf J. , Smet A. , Herman L. , Heyndrickx M. , Martel A. *et al* (2012) Antimicrobial use in Belgian broiler production. Preventive Veterinary Medicine 105, 320–5. 22459488 10.1016/j.prevetmed.2012.02.020

[vms389-bib-0043] Phuong Hua P.T. , Managaki S. , Nakada N. , Takada H. , Shimizu A. , Anh D.H. *et al* (2011) Antibiotic contamination and occurrence of antibiotic‐resistant bacteria in aquatic environments of northern Vietnam. Science of the Total Environment 409, 2894–2901. 21669325 10.1016/j.scitotenv.2011.04.030

[vms389-bib-0044] Rahmatallah N. , Nassik S. , EL Rhaffouli H. , Lahlou Amine I. , EL H.M. . (2013). Multiresistant avian pathogenic Escherichia coli isolated from broiler chickens in Rabat and regions in Morocco. Proceeding of the XVIII congress of the World Veterinary Poultry Association in Nantes, France. 19‐23 August. 2013.

[vms389-bib-0045] Rahmatallah N. , Nassik S. , El Rhaffouli H. , Lahlou Amine I. & El Houadfi M. (2017) Détection de souches multi‐résistantes d'Escherichia coli d'origine aviaire dans la région de Rabat‐Salé‐Zemmour‐Zaer. Revue Marocaine des Sciences Agronomiques et Vétérinaires 5, 96–102.

[vms389-bib-0046] Salama N.A. , Abou‐Raya S.H. , Shalaby A.R. , Emam W.H. & Mehaya F.M. (2011) Incidence of tetracycline residues in chicken meat and liver retailed to consumers. Food Additives & Contaminants. Part B, Surveillance 4, 88–93. 24785718 10.1080/19393210.2011.585245

[vms389-bib-0047] Timmerman T. , Dewulf J. , Catry B. , Feyen B. , Opsomer G. , de Kruif A. & Maes D. (2006) Quantification and evaluation of antimicrobial drug use in group treatments for fattening pigs in Belgium. Preventive Veterinary Medicine 74, 251–263. 16675051 10.1016/j.prevetmed.2005.10.003

[vms389-bib-0048] Wammer K.H. , Slattery M.T. , Stemig A.M. & Ditty J.L. (2011) Tetracycline photolysis in natural waters: loss of antibacterial activity. Chemosphere 85, 1505–10. 21959143 10.1016/j.chemosphere.2011.08.051

[vms389-bib-0049] Wegener H.C. (2003) Antibiotics in animal feed and their role in resistance development. Current Opinion in Microbiology 6, 439–445. 14572534 10.1016/j.mib.2003.09.009

[vms389-bib-0050] Werner J.J. , Arnold W.A. & McNeill K. (2006) Water hardness as a photochemical parameter: tetracycline photolysis as a function of calcium concentration, magnesium concentration, and pH. Environmental science & technology. 40, 7236–41. 17180972 10.1021/es060337m

[vms389-bib-0051] World Health Organisation (WHO) (2014). Report of the 1st meeting of the WHO advisory group on integrated surveillance of antimicrobial resistance, Copenhagen, 15‐19 June 2009. Available at: http://apps.who.int/iris/bitstream/10665/75199/1/9789241501446_eng.pdf?ua=1&ua=1. (Accessed on 01 March 2014)

[vms389-bib-0052] World Organisation For Animal Health (OIE) (2003) OIE guidelines on antimicrobial resistance: reports prepared by the OIE Ad hoc group of experts on antimicrobial resistance. Revue Scientifique et Technique (International Office of Epizootics) 20, 797–870.

[vms389-bib-0053] World Organisation For Animal Health (OIE) (2014). Terrestrial Animal Health Code. Available from : http://www.oie.int/index.php?id=169&L=0&htmfile=chapitre_antibio_monitoring.htm. (Accessed on 01 March 2014).

